# Visceral adiposity index performed better than traditional adiposity indicators in predicting unhealthy metabolic phenotype among Chinese children and adolescents

**DOI:** 10.1038/s41598-021-03311-x

**Published:** 2021-12-13

**Authors:** Yangyang Dong, Ling Bai, Rongrong Cai, Jinyu Zhou, Wenqing Ding

**Affiliations:** 1 School of Public Health and Management, Ningxia Medical University, No.1160, Shengli Street, Xingqing District, Yinchuan, Ningxia China; 2 Key Laboratory of Environmental Factors and Chronic Disease Control, Ningxia Medical University, No.1160, Shengli Street, Xingqing District, Yinchuan, Ningxia China

**Keywords:** Endocrinology, Medical research, Risk factors

## Abstract

The relationship between visceral adiposity index (VAI) and unhealthy metabolic phenotype remained unclear in children and adolescents. This study aimed to investigate their association and compared the ability of VAI and traditional adiposity indicators (body mass index, waist circumference and waist-to-height ratio) to predict metabolically unhealthy phenotype among normal-weight, overweight and obese children and adolescents. In this cross-sectional study, 1722 children and adolescents aged 12–18 years were selected by cluster random sampling, underwent a questionnaire survey, physical examination and biochemical tests. Participants were divided into four phenotypes according to the combination of the weight status determined by body mass index (BMI) and metabolic syndrome components. Receiver operating characteristic (ROC) analysis and multivariate logistic regression were used to compare the predictive capacity between VAI and traditional adiposity indicators and their relationship with metabolically unhealthy phenotype. We found that VAI had better performance in predicting metabolically unhealthy phenotype than traditional adiposity indicators, the area under the receiver-operating characteristic curve (AUC) were 0.808 and 0.763 for boys and girls with normal-weight, 0.829 and 0.816 for boys and girls with overweight and obese (all *P* < 0.001). VAI was most strongly related to metabolically unhealthy phenotype whether or not to adjust the age, the adjusted OR and 95%CI was 6.15 (4.13–9.14) in boys with normal weight, and 5.90 (3.06–11.36), 4.95 (2.35–10.41) in boys and girls with overweight and obese, respectively (all *P* < 0.001). Our findings suggested VAI could be used as a comprehensive predictor to identify unhealthy metabolic phenotype in children and adolescents.

## Introduction

The global prevalence of overweight and obesity among children and adolescents is increasing rapidly and has become a major public health problem^1^, especially in China^2^. Childhood obesity was associated with cardiac metabolic disorders, including elevated blood pressure (BP), hyperglycemia and hyperlipidemia^3,4^, and cardiovascular risk in children could be accompanied into adulthood, aggravating the risk of cardiac metabolism^5^. However, not all obese individuals had the same metabolic risk, on the contrary, they had favorable metabolic characteristics (such as high insulin sensitivity), which was defined metabolically healthy obesity^6,7^ and some individuals with normal-weight had metabolic disorders, which was defined metabolically unhealthy normal-weight (MUNW)^8^. Compared to metabolically healthy normal-weight (MHNW) individuals, metabolically unhealthy obesity and metabolically healthy overweight or obesity individuals had a higher risk of hypertension and cardiovascular disease^9–11^. Thus, early identification of different metabolic phenotypes was important for disease prevention among children and adolescents.

At present, the underlying mechanism for determining different metabolic phenotypes was unclear, which may be related to visceral fat accumulation^12^, inflammatory factors^13^, genetics^14^, dietary intake behaviors and lifestyle habits^15^. Some studies showed that visceral adipose tissue (VAT) was positively correlated with cardiometabolic risk factors^16^. As the gold standard for measuring visceral obesity, computed tomography and magnetic resonance imaging were characterized by radiation hazards and high costs, therefore, these techniques were not suitable for large-scale epidemiological investigations^17^. Visceral adiposity index (VAI), which combined anthropometric (body mass index (BMI) and waist circumference (WC)) and metabolic characteristics (serum triglyceride (TG) and high-density lipoprotein cholesterol (HDL-C)), was considered to be a reliable indicator of visceral fat^18^.

Studies suggested that compared with traditional adiposity indicators, such as WC and waist-to-height ratio (WHtR), VAI was most strongly related to the metabolically unhealthy phenotype, and showed better power of predicting unhealthy metabolic phenotype in adults^17,19^. However, the relationship between VAI and metabolically unhealthy phenotype and its predictive value remained unclear among children and adolescents.

Therefore, we aimed to explore the relationship between VAI and unhealthy metabolic phenotype and compare the ability of VAI and traditional adiposity indexes to identify unhealthy metabolic phenotype among normal-weight, overweight and obese children and adolescents, using data from China.

## Methods

### Study population

Using the method of cluster random sampling, children and adolescents aged 12 to 18 were randomly selected from 13 classes of 3 Junior Middle Schools and 10 classes of 2 Senior High Schools in Yinchuan city, China, from 2017 to 2020. They underwent a questionnaire survey, physical examination and laboratory examination. After excluding participants with missing information, a total of 1722 subjects were enrolled in this study. All subjects and their parents/guardians signed written informed consents. This study was approved by the Ethics Committee of Ningxia Medical University (2016-123) and conducted in accordance with the Declaration of Helsinki.

### Questionnaire investigation

The content of the questionnaire included the basic information of the subjects (name, sex, age and date of birth), dietary habits (smoking, drinking and diet) and so on. The basic information of the subjects and the high fat food consumption were used for data analysis. High fat food consumption defined as consumption of at least one meat, fried food or western fast food a week, including poultry meat, Youtiao, fried chicken, hamburger and so on.

### Biochemical measurements

After an overnight fast of 12 h, venous blood samples were obtained into the procoagulant tube and centrifuged by 3000 r/min for 15 min after resting for 30 min; then the serum was collected and stored in the refrigerator at – 80 °C. The levels of total cholesterol (TC), TG, HDL-C, low-density lipoprotein cholesterol (LDL-C) and fasting plasma glucose (FPG) were determined using the American AU480 automatic biochemical analyzer.

### Anthropometrical data

All anthropometric variables were measured by trained staff in strict accordance with the standard scheme. Height and WC were measured twice for subjects in light clothes without shoes to the nearest 0.1 cm, WC was measured at the midway between the inferior margin of the last rib and iliac crest at the end of normal exhalation, and weight also was measured twice to the nearest 0.1 kg with a lever scale (RGT-140, Wuxi Weigher Factory Co.,Ltd, China), the error of two measurements was no more than 0.5 cm or 0.5 kg. Mean value of weight and height were used to calculate BMI and WHtR. BMI = weight (kg)/height^2^ (m^2^), WHtR = WC (cm)/height (cm). For boys, VAI = [WC/(39.68 + 1.88 × BMI)] × (TG/1.03) × (1.31/HDL-C), for girls, VAI = [WC/(36.58 + 1.89 × BMI)] × (TG/0.81) × (1.52/HDL-C), with TG and HDL-C levels expressed as mmol/l. According to the method recommended by the American Hypertension Education Project working Group^20^, seated systolic blood pressure (SBP) and diastolic blood pressure (DBP) were measured continuously for 3 times with at least 1 min between repeated measurements, using an electronic sphygmomanometer (HEM-7012, Omron, Japan), and the difference between the two adjacent measurements was no more than 10 mmHg. The average value of the last two readings was used for analysis.

### Definitions

The participants were divided into four metabolically phenotypes as following: (1) metabolically healthy normal-weight (MHNW); (2) metabolically unhealthy normal weight (MUNW); (3) metabolically healthy overweight or obesity (MHO); (4) metabolically unhealthy overweight or obesity (MUO). We used the presence of metabolic syndrome (MetS) components to define metabolic abnormalities, as follows^21^: (1) WC ≥ 95th percentile for age and sex^22^; (2) TG levels ≥ 1.24 mmol/L; (3) HDL-C ≤ 1.03 mmol/L; (4) SBP and/or DBP ≥ 90th percentile for age, sex and height^23^; (5) FPG levels ≥ 5.6 mmol/L. Individuals who meet at least two of the criteria mentioned above were considered metabolically unhealthy. According to the recommendation of the Working Group on obesity (WGOC) in China^24^, BMI was used to define normal-weight, overweight and obesity in children and adolescents.

### Statistical analysis

Continuous variables were expressed as mean ± standard deviation (SD) or median (25th–75th percentiles), and the differences between groups were compared using *t*-test or the Wilcoxon rank sum test of two independent samples. Categorical variables were presented as percentage, and the chi-square test was used to compare the differences between groups. The Z-scores by age and sex of VAI, BMI, WC and WHtR were calculated and used for receiver operating characteristic (ROC) analysis and multivariate logistic regression, Z-score = (test value-mean value by age and gender) /SD by age and gender. ROC analysis was used to compare the ability of VAI, BMI, WC and WHtR to predict MUNW and MUO; we evaluated the association of VAI, BMI, WC and WHtR with metabolic phenotypes by multivariate logistic regression, with adjustment for age. Epidata3.1 software was used to input data, and data were analyzed using SPSS26.0. Two-sided *P* < 0.05 was considered to be statistically significant.

## Results

Among the 1722 children and adolescent evaluated, the prevalence of MHNW MUNW, MHO and MUO was 65.9%, 12.2%, 5.9% and 16.1%, respectively. Compared to girls, boys showed a lower prevalence of MUNW (9.3% vs 16.9%, *P* < 0.001), but there was no difference in the prevalence of MUO between the two groups (16.8% vs 14.9%, *P* = 0.290).

Tables 1 and 2 presented characteristics of subjects according to different phenotypes. Among normal-weight children and adolescents, the prevalence of MUNW was 15.6%, compared with girls, boys had a lower prevalence of MUNW (12.1% vs 21.1%, *P* < 0.001). Among overweight and obese children and adolescents, the prevalence of MUO was 73.3%, and no difference was found in the prevalence of MUO between boys and girls (72.8% vs 74.2%, *P* = 0.757). Compared to their metabolically healthy counterparts, metabolically unhealthy subjects showed higher weight, BMI, WC, WHtR, SBP, DBP, TG, FPG and VAI in both normal-weight, overweight and obese children and adolescents (all *P* < 0.05). In addition, MUNW individuals were older and had higher TC and LDL-C than those with MHNW; MUO individuals had higher height and lower HDL-C than MHO individuals (all *P* < 0.05). After gender stratification analysis, the results were consistent with the whole population, but no significant differences in FPG were found between MHO and MUO individuals in both genders, there were no significant differences in weight, BMI, WC and WHtR between MHO and MUO girls. Significant differences in consumption of high fat food were only found between MHNW and MUNW boys (*P* < 0.05).

**Table 1:** Characteristics of subjects according to different phenotypes among normal-weight children and adolescents.

Variables	Total (n = 1344)		*P* value	Boys (n = 818; 60.9%)		*P* value	Girls (n = 526; 39.1%)		*P* value
	MHNW	MUNW		MHNW	MUNW		MHNW	MUNW	
n (%)	1134 (84.4)	210 (15.6)		719 (87.9)	99 (12.1)		415 (78.9)	111 (21.1)	
Age (years)	15.0 ± 1.6	15.4 ± 1.5	0.001	15.3 ± 1.5	15.6 ± 1.3	0.024	14.7 ± 1.7	15.3 ± 1.6	0.001
Height (cm)	167.5 ± 8.5	168.3 ± 8.3	0.238	170.7 ± 8.1	174.0 ± 6.67	< 0.001	161.9 ± 5.9	163.1 ± 5.9	0.057
Weight (kg)	53.0 ± 7.7	57.3 ± 7.9	< 0.001	54.9 ± 7.9	59.7 ± 8.5	< 0.001	49.6 ± 5.8	55.1 ± 6.7	< 0.001
BMI (kg/m^2^)	18.84 ± 1.87	20.24 ± 2.10	< 0.001	18.78 ± 1.86	19.75 ± 2.13	< 0.001	18.94 ± 1.87	20.68 ± 1.99	< 0.001
WC (cm)	69.56 ± 4.90	74.31 ± 6.05	< 0.001	69.15 ± 4.87	73.16 ± 6.70	< 0.001	70.27 ± 4.87	75.35 ± 5.23	< 0.001
WHtR	0.42 ± 0.03	0.44 ± 0.04	< 0.001	0.41 ± 0.03	0.42 ± 0.03	< 0.001	0.43 ± 0.03	0.46 ± 0.03	< 0.001
SBP (mmHg)	109.1 ± 9.8	114.1 ± 12.2	< 0.001	110.6 ± 10.1	118.1 ± 12.1	< 0.001	106.5 ± 8.5	110.5 ± 11.2	0.001
DBP (mmHg)	66.5 ± 7.3	71.1 ± 8.6	< 0.001	65.9 ± 7.7	70.1 ± 8.7	< 0.001	67.4 ± 6.5	72.0 ± 8.4	< 0.001
TC (mmol/L)	3.87 ± 0.87	4.19 ± 1.10	< 0.001	3.84 ± 0.84	4.16 ± 1.15	0.008	3.93 ± 0.92	4.22 ± 1.06	0.010
TG (mmol/L)	0.86 (0.69–1.04)	1.32 (0.91–1.57)	< 0.001	0.83 (0.68–1.03)	1.35 (1.01–1.60)	< 0.001	0.88 (0.71–1.06)	1.31 (0.85–1.54)	< 0.001
HDL-C (mmol/L)	1.46 ± 0.35	1.44 ± 0.56	0.513	1.44 ± 0.33	1.37 ± 0.53	0.177	1.49 ± 0.38	1.50 ± 0.57	0.977
LDL-C (mmol/L)	2.10 ± 0.70	2.30 ± 0.83	0.001	2.10 ± 0.70	2.29 ± 0.91	0.052	2.07 ± 0.70	2.30 ± 0.77	0.006
FPG (mmol/L)	4.69 ± 0.60	5.25 ± 1.04	< 0.001	4.70 ± 0.65	5.56 ± 1.16	< 0.001	4.66 ± 0.52	4.97 ± 0.83	0.001
VAI	0.81 (0.61–1.10)	1.47 (0.94–2.10)	< 0.001	0.69 (0.54–0.88)	1.22 (0.85–1.77)	< 0.001	1.10 (0.86–1.37)	1.66 (1.12–2.38)	< 0.001
High fat food consumption (n, %)^a^	674 (88.9)	126 (90.6)	0.546	411 (88.8)	63 (96.9)	0.042	2 (89.2)	63 (85.1)	0.336

Normally distributed data are expressed as the mean ± standard deviation (SD), skewed variables are expressed as the median (25th-75th percentiles).

*MHNW* metabolically healthy normal-weight, *MUNW* metabolically unhealthy normal-weight, *BMI* body mass index, *WC* waist circumference, *WHtR* waist-to-height ratio, *SBP* systolic blood pressure, *DBP* diastolic blood pressure, *TC* total cholesterol, *TG* triglycerides, *HDL-C* high-density lipoprotein cholesterol, *LDL-C* low-density lipoprotein cholesterol, *FPG* fasting plasma glucose, *VAI* visceral adiposity index.

^a^Consumption of at least one meat, fried food or western fast food a week.

**Table 2:** Characteristics of subjects according to different phenotypes among overweight and obese children and adolescents.

Variables	Total (n = 378)		*P* value	Boys (n = 246; 65.1%)		*P* value	Girls (n = 132; 34.9%)		*P* value
	MHO	MUO		MHO	MUO		MHO	MUO	
n (%)	101 (26.7)	277 (73.3)		67 (27.2)	179 (72.8)		34 (25.8)	98 (74.2)	
Age (years)	14.5 ± 1.5	14.6 ± 1.6	0.338	14.3 ± 1.5	14.7 ± 1.5	0.080	14.7 ± 1.5	14.4 ± 1.5	0.432
Height (cm)	167.7 ± 8.1	170.5 ± 8.3	0.004	170.6 ± 7.6	173.8 ± 7.4	0.003	162.1 ± 5.9	164.6 ± 6.3	0.046
Weight (kg)	70.9 ± 10.8	78.0 ± 12.5	< 0.001	72.5 ± 11.6	81.8 ± 12.3	< 0.001	67.6 ± 8.2	71.2 ± 9.8	0.058
BMI (kg/m^2^)	25.14 ± 2.68	26.76 ± 2.93	< 0.001	24.83 ± 2.70	27.02 ± 3.00	< 0.001	25.76 ± 2.56	26.28 ± 2.75	0.334
WC (cm)	84.68 ± 8.25	90.83 ± 9.38	< 0.001	83.63 ± 8.83	92.01 ± 9.81	< 0.001	85.64 ± 6.89	88.66 ± 8.15	0.055
WHtR	0.50 ± 0.05	0.53 ± 0.05	< 0.001	0.49 ± 0.04	0.53 ± 0.05	< 0.001	0.53 ± 0.04	0.54 ± 004	0.235
SBP (mmHg)	112.0 ± 8.1	123.2 ± 10.3	< 0.001	113.9 ± 7.8	124.6 ± 10.5	< 0.001	108.3 ± 7.7	120.7 ± 9.5	< 0.001
DBP (mmHg)	65.6 ± 5.6	72.8 ± 8.3	< 0.001	66.1 ± 5.7	71.9 ± 8.1	< 0.001	64.6 ± 5.4	74.4 ± 8.5	< 0.001
TC (mmol/L)	3.92 ± 0.82	4.12 ± 1.05	0.056	3.87 ± 0.80	4.11 ± 1.10	0.055	4.04 ± 0.85	4.13 ± 0.95	0.593
TG (mmol/L)	0.85 (0.71–1.08)	1.31 (0.96–1.69)	< 0.001	0.88 (0.69–1.10)	1.32 (0.97–1.67)	< 0.001	0.82 (0.71–1.04)	1.30 (0.94–1.70)	< 0.001
HDL-C (mmol/L)	1.38 ± 0.31	1.24 ± 0.31	< 0.001	1.32 ± 0.28	1.19 ± 0.30	0.003	1.49 ± 0.34	1.33 ± 0.33	0.017
LDL-C (mmol/L)	2.21 ± 0.70	2.37 ± 0.94	0.119	2.22 ± 0.68	2.42 ± 1.00	0.067	2.18 ± 0.74	2.26 ± 0.81	0.627
FPG (mmol/L)	4.70 ± 0.44	4.83 ± 0.62	0.031	4.75 ± 0.45	4.87 ± 0.67	0.127	4.61 ± 0.43	4.77 ± 0.51	0.116
VAI	0.89 (0.73–1.21)	1.53 (1.11–2.31)	< 0.001	0.86 (0.63–1.09)	1.40 (1.06–2.05)	< 0.001	1.05 (0.82–1.56)	1.96 (1.29–2.73)	< 0.001
High fat food consumption (n, %)^a^	55 (87.3)	188 (87.9)	0.907	37 (97.4)	120 (91.6)	0.390	18 (72.0)	68 (81.9)	0.280

Normally distributed data are expressed as the mean ± standard deviation (SD), skewed variables are expressed as the median (25th-75th percentiles).

*MHO* metabolically healthy overweight or obese, *MUO* metabolically unhealthy overweight or obese, *BMI* body mass index, *WC* waist circumference, *WHtR* waist-to-height ratio, *SBP* systolic blood pressure, *DBP* diastolic blood pressure, *TC* total cholesterol, *TG* triglycerides, *HDL-C* high-density lipoprotein cholesterol, *LDL-C* low-density lipoprotein cholesterol, *FPG* fasting plasma glucose, *VAI* visceral adiposity index.

^a^Consumption of at least one meat, fried food or western fast food a week.

Table 3 and Supplementary Fig. 1 and Fig. 2 showed the ROC curve analysis of the VAI and traditional adiposity indicators for predicting unhealthy metabolic phenotype among normal-weight, overweight and obese individuals. VAI, BMI, WC and WHtR were able to predict MUNW and MUO in the whole population, and both sexes, except BMI and WHtR in overweight and obese girls. VAI presented the greatest diagnostic precision for predicting MUNW in participants with normal-weight (AUC of the whole population, boys and girls were 0.784, 0.808 and 0.763, respectively) and MUO in individuals with overweight and obesity (AUC of the whole population, boys and girls were 0.822, 0.829 and 0.816, respectively, all *P* < 0.05). In the traditional adiposity indicators, WC showed better ability to predict unhealthy metabolic phenotype in both genders. For boys and girls, the AUC were 0.667 and 0.746 in predicting MUNW, 0.761 and 0.618 in predicting MUNW phenotype, respectively (all *P* < 0.05).

**Table 3:** ROC curve analysis of the VAI and traditional adiposity indicators for predicting metabolically unhealthy phenotype among normal-weight, overweight and obese individuals.

Test variables	MUNW			MUO		
	AUC ± SE	95% CI	*P* value	AUC ± SE	95% CI	*P* value
**Total**						
VAI	0.784 ± 0.020	0.745–0.824	< 0.001	0.822 ± 0.022	0.779–0.864	< 0.001
BMI (kg/m^2^)	0.682 ± 0.021	0.641–0.724	< 0.001	0.694 ± 0.032	0.631–0.757	< 0.001
WC (cm)	0.709 ± 0.021	0.668–0.749	< 0.001	0.716 ± 0.031	0.656–0.776	< 0.001
WHtR	0.685 ± 0.021	0.645–0.728	< 0.001	0.692 ± 0.031	0.631–0.753	< 0.001
**Boys**						
VAI	0.808 ± 0.027	0.755–0.862	< 0.001	0.829 ± 0.026	0.777–0.880	< 0.001
BMI (kg/m^2^)	0.632 ± 0.031	0.572–0.692	< 0.001	0.746 ± 0.037	0.674–0.818	< 0.001
WC (cm)	0.667 ± 0.029	0.610–0.725	< 0.001	0.761 ± 0.036	0.692–0.831	< 0.001
WHtR	0.626 ± 0.031	0.564–0.687	< 0.001	0.743 ± 0.036	0.673–0.813	< 0.001
**Girls**						
VAI	0.763 ± 0.029	0.706–0.820	< 0.001	0.816 ± 0.039	0.739–0.892	< 0.001
BMI (kg/m^2^)	0.730 ± 0.029	0.674–0.787	< 0.001	0.584 ± 0.058	0.470–0.697	0.147
WC (cm)	0.746 ± 0.029	0.690–0.802	< 0.001	0.618 ± 0.057	0.506–0.730	0.041
WHtR	0.738 ± 0.028	0.683–0.793	< 0.001	0.583 ± 0.058	0.469–0.696	0.152

Table 4 presented the result of multivariate logistic regression analysis. Regardless of whether the analytical model adjusted age or not, VAI and WC were associated with MUNW and MUO. VAI had a stronger association with MUNW and MUO, the adjusted OR and 95% confidence interval (CI) were 4.45 (3.45–5.73) and 5.11 (3.16–8.26), respectively (all *P* < 0.05). There were similar findings among boys, the adjusted OR and 95%CI were 6.15 (4.13–9.14) and 5.90 (3.06–11.36), respectively (all *P* < 0.05). However, the association of VAI and BMI with MUNW were found among normal-weight girls, and BMI showed a stronger association, age-adjusted OR and 95%CI was 4.16 (2.53–6.82) (*P* < 0.05); VAI was the independent determinants of MUO among overweight or obesity girls, the age-adjusted OR and 95%CI was 4.95 (2.35–10.41) (*P* < 0.05).

**Table 4:** Multivariate logistic regression analysis for associations of the visceral adiposity index and traditional adiposity indicators with metabolically unhealthy phenotype among normal-weight, overweight and obese volunteers.

	Total		Boys		Girls	
	OR (95% CI)	Age-adjusted OR (95% CI)	OR (95% CI)	Age-adjusted OR (95% CI)	OR (95% CI)	Age-adjusted OR (95% CI)
**MUNW**						
VAI	4.45 (3.45–5.73)^a^	4.45 (3.45–5.73)^a^	6.15 (4.13–9.14)^a^	6.15 (4.13–9.14)^a^	3.54 (2.58–4.84)^a^	3.50 (2.55–4.81)^a^
BMI (kg/m^2^)	1.74 (1.00–3.03)^c^	1.74 (1.00–3.03)^c^	–	–	4.27 (2.62–6.98)^a^	4.16 (2.53–6.82)^b^
WC (cm)	1.94 (1.11–3.37)^b^	1.94 (1.11–3.37)^b^	2.21 (1.35–3.60)^b^	2.21 (1.35–3.60)^b^	–	–
WHtR	–	–	–	–	–	–
**MUO**						
VAI	5.11 (3.16–8.26)^a^	5.11 (3.16–8.26)^a^	5.90 (3.06–11.36)^a^	5.90 (3.06–11.36)^a^	4.95 (2.35–10.41)^a^	4.95 (2.35–10.41)^a^
BMI (kg/m^2^)	–	–	–		–	–
WC (cm)	1.94 (1.33–2.85)^b^	1.94 (1.33–2.85)^b^	2.34 (1.40–3.90)^b^	2.34 (1.40–3.90)^b^	–	–
WHtR	–	–	–		–	–

Used step forward regression. ^a^*P* < 0.001, ^b^*P* < 0.05, ^c^*P* = 0.05.

## Discussion

Our results indicated that among children and adolescents, compared to traditional adiposity indexes (BMI, WC and WHtR), VAI had the greatest power to identify the unhealthy metabolic phenotype and VAI was most strongly related to the MUNW and MUO phenotype. After gender stratification analysis, the results were similar, but except that BMI was most strongly associated with the MUNW phenotype in normal-weight girls. In addition, WC performed better than BMI and WHtR in predicting unhealthy metabolic phenotype in both sexes.

Due to the difference in diagnostic criteria for obesity and metabolic abnormalities, age range and population source, the prevalence of metabolically unhealthy phenotype was different in children and adolescents or adults. Some research suggested that among children and adolescents, the prevalence of MUNW and MUO ranged from 10.6–18.8%^25–27^ to 1.3–15.3%, respectively^25,26,28–30^. Our results were roughly consistent with the above research (12.2% and 16.1%, respectively). What’s more, compared with their metabolically healthy counterparts, individuals with MUNW and MUO showed higher VAI, BMI, WC, WHtR and worse metabolic profiles, which agreed with previous studies^19,26^.

A cross-section study for Chinese adults suggested that VAI and lipid accumulation product (LAP) were effective markers for identifying MUNW phenotype, the AUC of VAI ranged from 0.611 to 0.835 based on the criteria used for MUNW phenotype^17^. Data from Brazil indicated that VAI had the better predictive capacity and stronger association with metabolically unhealthy phenotype than other adiposity indexes (BMI, WHtR, waist-to-hip ratios, WC and neck circumference), the AUC for MUNW were 0.865 and 0.847 for men and women with normal weight, the AUC for MUO were 0.830 and 0.903 for men and women with overweight^19^. Ahmad reported that VAI proved to be superior to other obesity indicators in predicting cardiometabolic risks, VAI showed the largest AUC for men and women (0.79 vs 0.77), followed by waist-to-hip ratios (0.73 vs 0.75), WC (0.69 vs 0.74), WHtR (0.65 vs 0.71) and BMI (0.53 vs 0.51), respectively^31^. A cohort study followed up for ten years demonstrated that MHO was a transient state, with higher conversion to MUO independently related to VAT, higher fasting insulin level, et al^32^. Vizzuso found that VAI could be a promising tool to identify MetS in children and adolescents with obesity^33^. These findings were consistent with our results; VAI showed the strongest prediction ability, it may be due to VAI served as a powerful surrogate marker of visceral adiposity, while these traditional obesity indicators could only provide limited fat distribution information and lack the ability to distinguish between visceral and subcutaneous fat^34^. These findings suggested that VAI could be a better surrogate index than single traditional adiposity indices to evaluate the adverse metabolic phenotype of children and adolescents. The levels of visceral fat mass were positively associated with elevated BP and dyslipidemia among normal-weight, overweight and obese children and adolescent, which supported our view^25^.

However, a cohort of obesity children and adolescents aged 4 to 18 evaluated the impact of WHtR, serum Uric Acid and Homeostatic Model Assessment index of insulin resistance on the probability to be MUO, and found that these indexes were independent predictors of the probability of being MUO^35^. Data from the US National Health and Nutrition Examination Surveys (NHANES, 1999–2012) demonstrated that the AUC of BMI-z score, WHtR-z score and WC-z score to detect at least three cardiovascular risk factors ranged from 0.84 to 0.85 either using the International Diabetes Federation (IDF) or the revised National Cholesterol Education Program Adult Treatment Panel III (ATP III) criteria^36^. The results of these studies were inconsistent with ours, it may be explained by different standards of metabolic abnormalities, demographic characteristics and variables analyzed.

Chinese visceral adiposity index (CVAI), a novel indicator of visceral obesity, was developed in Chinese adults when considering ethnic differences in body fat characteristics^37,38^. CVAI increased the factor of the demographic (age) based on VAI^39^. Some studies indicated that CVAI and its 6-year change were positively related to hypertension risk, CVAI was superior to VAI, BMI, WC and a body shape index (ABSI) in predicting hypertension for both genders^40^, and CVAI had better performance in predicting diabetes than BMI, WC and ABSI in both sexes^41^. These results suggested that compared with VAI, CVAI may be more suitable and reliable for predicting metabolically unhealthy phenotype in Chinese adults. Studies from China indicated that the VAT of both boys and girls aged 6–18 years old showed a wavy increase trend with the increase of age^42^. However, the content of VAT in men firstly increased and then decreased with age, and reached the peak at 30 years old, the VAT content of women increased rapidly from 20 to 50 years old and peaked at 50 years old, after 50 years old, it gradually decreased and stabilized^43^. Due to the different trend for age of VAT between children and adults, whether CVAI could be used as an effective indicator of VAT in children need to be verified among a large sample population.

At present, the underlying mechanism of VAI and abnormal metabolic phenotype remained unclear. The possible mechanisms were as follows. First, individuals with higher VAT had higher levels of inflammatory cytokines, such as tumour necrosis factor-α, C-reactive protein and interleukin-6, which may lead to insulin resistance and metabolic disorders^44^. The ectopic fat deposition was also a possible cause^45^. Second, the increase of VAT was related to the decrease of circulating concentrations of B-type natriuretic peptide, which may diminish natriuresis and vasodilation and lead to hypertension^46^. In addition, we found WC was positively associated with metabolically unhealthy phenotype in boys, it could be explained by that WC was correlated with increased free fat acid and adipokines, increased oxidative stress, higher activity of inflammation and blunt insulin sensitivity^47^. We also found that BMI was more strongly correlated with MUNW phenotype than VAI in girls, which may be partly ascribed to the fact that female’s fat mainly accumulated under the skin^48^, while in girls with normal-weight, the subcutaneous fat might have a greater effect on metabolism than VAT.

Our research had the following limitations. First, the cross-sectional study made it impossible for us to explore the causal relationship between VAI and metabolically unhealthy phenotype. Second, the subjects of our study were limited to children and adolescents aged 12–18 in Yinchuan city, China, and the results may not be generalisable to other populations. Third, confounding factors including physical activity and lifestyle were not assessed in this study, which might have influenced our results. Fourth, our findings may vary depending on the different definition of metabolic phenotype. Nevertheless, we compared the ability of VAI and traditional obesity indicators to identify adverse metabolic phenotypes, so as to provide the scientific basis for the prevention of metabolic diseases in children and adolescents.

## Conclusions

In conclusion, the study indicated that compared with traditional adiposity indicators, VAI had the better power of predicting metabolically unhealthy phenotype in both genders, and VAI was most strongly associated with metabolically unhealthy phenotype in boys and MUO phenotype in overweight and obese girls, regardless of whether the age was adjusted or not. VAI might be used as a comprehensive index for predicting adverse metabolic phenotype in children and adolescents. What’s more, in the traditional adiposity indicators, WC was the better index for the screening of metabolically unhealthy phenotype in both genders.

## Supplementary Information

Supplementary Information 1.

Supplementary Information 2.
