# Supplementary material for: Visceral adiposity index performed better than traditional adiposity indicators in predicting unhealthy metabolic phenotype among Chinese children and adolescents
Source: Sci Rep. 2021 Dec 13;11:23850. doi: 10.1038/s41598-021-03311-x (PMC8668984; doi:10.1038/s41598-021-03311-x)
Supplement: Supplementary file 1 — Supplementary Information 1. [file 41598_2021_3311_MOESM1_ESM.docx]

Supplementary material


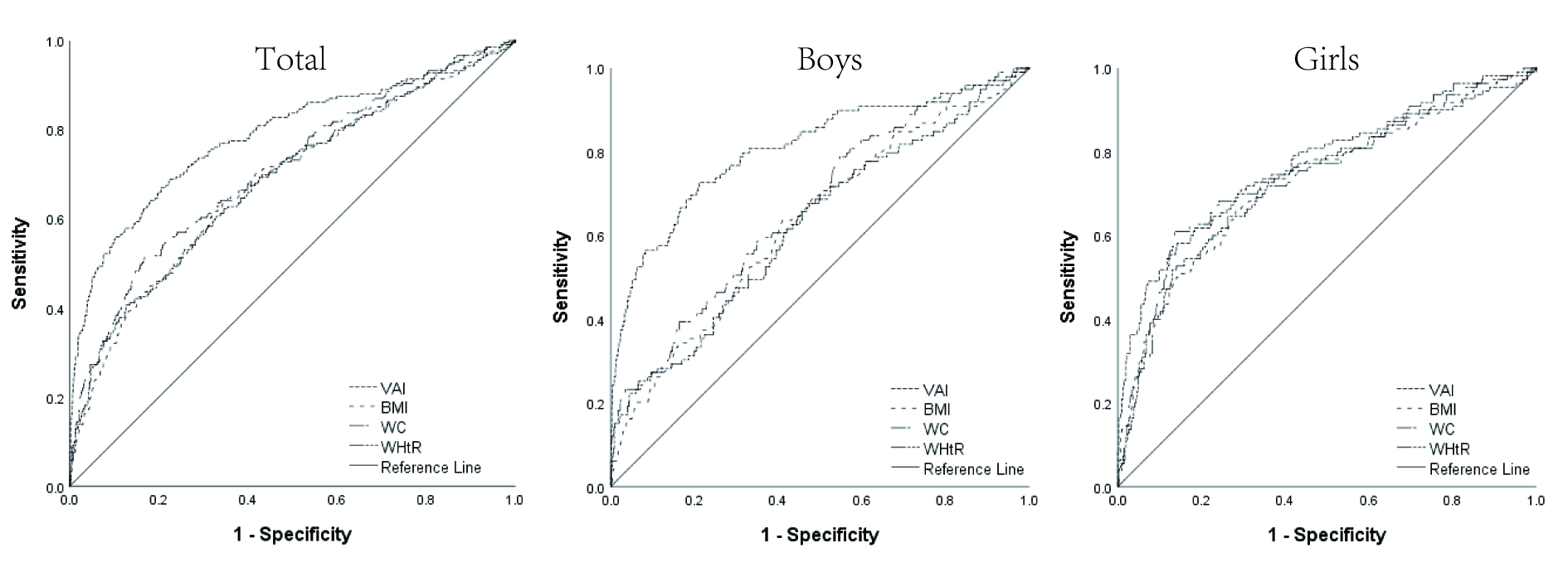


Figure 1 ROC curves of VAI, BMI, WC and WHtR to predict MUNW phenotype among normal-weight children and adolescents.


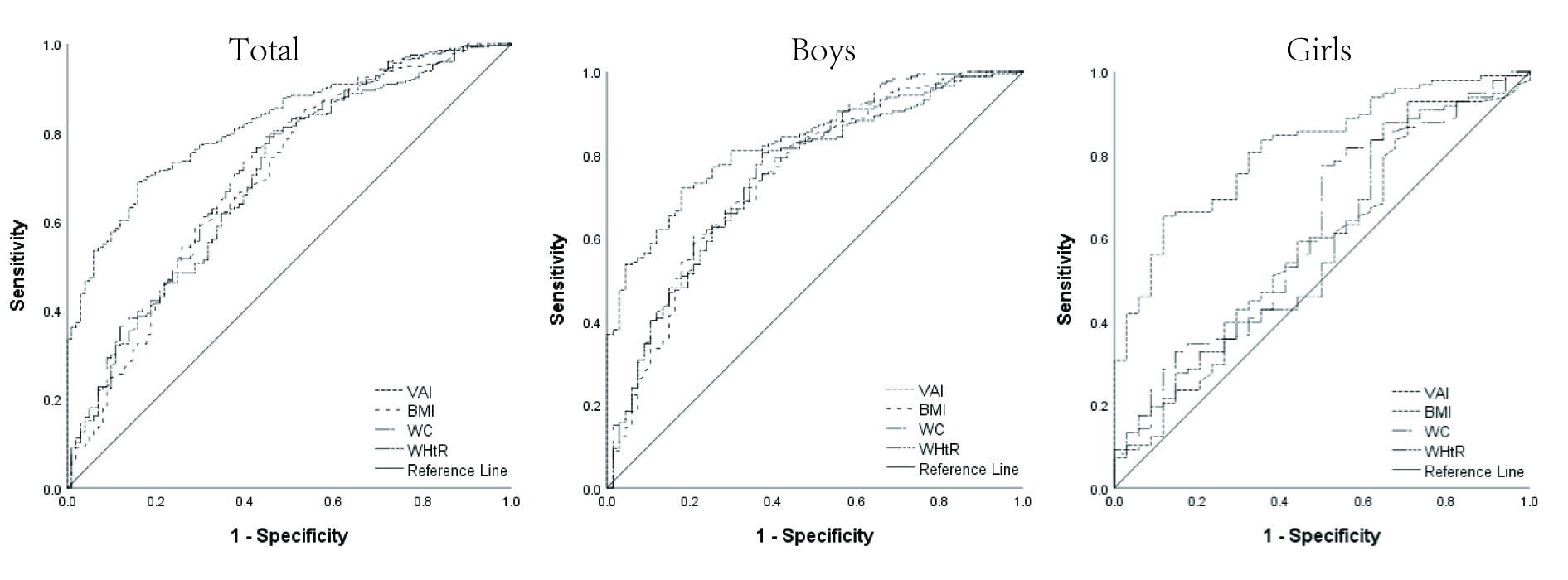


Figure 2 ROC curves of VAI, BMI, WC and WHtR to predict MUO phenotype among overweight and obese children and adolescents.
