# Supplementary material for: Visceral adiposity index performed better than traditional adiposity indicators in predicting unhealthy metabolic phenotype among Chinese children and adolescents
Source: Sci Rep. 2021 Dec 13;11:23850. doi: 10.1038/s41598-021-03311-x (PMC8668984; doi:10.1038/s41598-021-03311-x)
Supplement: Supplementary file 2 — Supplementary Information 2. [file 41598_2021_3311_MOESM2_ESM.pdf]

Investigation on Blood pressure of Children in Yinchuan City, China--Standard questionnaire

serial number ☐☐☐☐☐☐

A.Basic situation

A1 **Name**_______________ **Telephone**____________

A2 **Gender** 1. Male 2. Female — A2 ☐

A3 **Date of birth**_______

A4 **Ethnic group** — A4 ☐
1.Han nationality 2.Manchu nationality 3.Hui nationality 4.Other

A4m **The nation of the mother of the child** — A4m ☐
1.Han nationality 2.Manchu nationality 3.Hui nationality 4.Other

A4f **The nation of the father of the child** — A4f ☐
1.Han nationality 2.Manchu nationality 3.Hui nationality 4.Other

A5 **School** __________________________ **Grade**__________ **Class**_______

A6 **Name of father**_____________ **Telephone**_______

A7 **Name of mother**_____________ **Telephone**_______

A8 **Home address** _______________ **Postal code**_______

B.Birth and infantile feeding

B1 **Whether it is a singleton or not** 0.No 1.Yes — B1 ☐

B2 **Birth condition** — B2 ☐
1.Preterm delivery (less than 37 gestational weeks)
2.Full-term delivery (37-42 gestational weeks)
3.Overdue delivery (≥ 42 gestational weeks)
9.Unknown

B3 **Birth weight**_____________________Kg

B4 **Feeding in the first 6 months of life**
1.Exclusive breast milk
2.Basic breast milk (3 beat 4 or more)
3.Breast milk accounts for half
4.A small amount of breast milk (1 beat 4)
5.Completely non-breast milk
9.Unknown — B4 ☐

C.Growth and development

Filled in by girls

C1 **Menstruation** — C1 ☐
0.No menstruation
1.Already menstruated but irregular
2.Have regular menstruation
9.Unknown

C2 **Time of first menstruation**___________ — C2 ☐☐☐☐☐☐

or_______**Years old** ☐☐

Filled in by boys

C3 **Spermatorrhea of boys** — C3 ☐
0.Exhaustive semen 1.Spermatorrhea 9.Unknown

C4 **Time of first Spermatorrhea**___________ — C2 ☐☐☐☐☐☐

or_______**Years old** ☐☐

D.Diet and Living Habits

D1 **Are you a vegetarian?** 0.No 1. partial vegetarian 2.completel vegetarian — D1 ☐

D2 ☐

D2 **Have you often eaten breakfast in the past six months?**

0.No 1.Yes

D3 ☐

D3 **Smoking situation**
0.Never smoke
1.Try smoking (try smoking, even if only one or two mouthfuls)
2.Recent smoking (at least one cigarette in the past 30 days)

D4 ☐

D4 **Alcohol consumption**
0.Never drink alcohol
1. Try to drink alcohol (at least half a bottle or a beer, a small glass of white wine, etc)
2.Drink now (at least one drink in the past 30 days)
3.Heavy drinking (5 drinks in 2 hours on at least one of the last 30 days)
4.Drunkenness (drunkenness symptoms such as dizziness / headache / drowsiness due to excessive drinking in the past 12 months)

D5 M**eal survey the frequency of eating the following foods in the past 12 months?**(Please fill in only one answer for each type of food. If you do not eat, please fill in the last column 0.If you eat less than once a day, please fill in how many times a week; If the frequency is less than once a week, please fill in how many times a month)

	Number of times/Day	Number of times/Week	Number of times/Month	No food
Meat/fowl (pigs, cattle, sheep, chickens, ducks, geese)	☐	☐	☐	☐
Aquatic products (fish, shrimp, shellfish)	☐	☐	☐	☐
Eggs	☐	☐	☐	☐
Coarse grain (corn, millet, purple rice, sorghum)	☐	☐	☐	☐
Fine grain (fine rice, white flour and its products)	☐	☐	☐	☐
Milk and dairy products (fresh milk, milk powder, cheese)	☐	☐	☐	☐
Fried food/Foreign fast food (sticks, fried chicken, hamburgers, etc)	☐	☐	☐	☐
Soy sauce/Sauce products (soy sauce, noodle sauce, meat sauce)	☐	☐	☐	☐
Pickled vegetables and pickled products (pickled vegetables, bacon, salted eggs)	☐	☐	☐	☐
Salty snacks (French fries, potato chips, biscuits)	☐	☐	☐	☐
High-sugar foods (jam, cake, chocolate, candy, dessert, cola, juice, etc.)	☐	☐	☐	☐
Sugar (white sugar, brown sugar, honey, syrup)	☐	☐	☐	☐
Soybean products (soy milk, tofu, bean sprouts, peas, beans)	☐	☐	☐	☐
Nuts (walnuts, almonds, chestnuts, melon seeds, peanuts, etc.)	☐	☐	☐	☐
Sugary carbonated drinks (soda, cola, Sprite, etc.)<br>Note: drink intake write down how many milliliters of (ml) to drink each time.	☐ml/Day	☐ml/Week	☐ml/Month	☐
Fruit juice drinks (excl. pure fruit juices)	☐ml	☐ml	☐ml	☐
Fruit / pure fruit juice	☐	☐	☐	☐
Leafy vegetables (spinach, cabbage, rape, etc.)	☐	☐	☐	☐
Non-leafy vegetables (raw)	☐	☐	☐	☐

Non-leafy Vegetables (cooked)				☐
Calcium supplement preparation (such as calcium tablet, calcium oral liquid, etc.)	☐	☐	☐	☐
Vitamin D supplements (such as cod liver oil, vitamin D capsules, etc.)	☐	☐	☐	☐

E.**Physical activity and sleep (in the last half year)**

E1 **What are the main means of transportation for going to and from school?**

1.Walk
2.Cycling
3.Take a private car or bike
4.By bus

E1 ☐

E2 **The means of transportation and time used for going to and from school** (only counting activities that last more than 10 minutes each time, you can choose more than one)

E2-1 Cycling ______days / weeks; An average of ______ hours and ______minutes per day

E2-2 Walk ______days / weeks; An average of ______ hours and ______minutes per day

E2-3 Take a private car or bike ______days / weeks; An average of ______hours and ______minutes per day

E2-4 By bus ______days / weeks; An average of ______ hours and ______minutes per day

E3 **On a weekly basis, the time you spend on the following physical exercises is (only counting activities that last more than 10 minutes at a time)**

E3-1 High-intensity physical exercise (medium-speed running, medium-speed swimming, football, basketball, badminton, etc.)
______days / weeks; An average of ______ hours and ______minutes per day

E3-2 Medium intensity physical exercise (fast walking, jogging, slow swimming, Taijiquan, table tennis, fan dance, ballroom dance, Yangko, etc.)
______days / weeks; An average of ______ hours and ______minutes per day

E3-3 Ordinary walking______days / weeks; An average of ______ hours and ______minutes per day

E4 **Average daily time spent on the following activities (watching TV, using the computer, playing games, reading)** ______ hours and ______minutes

E5-1 **When you are at school, you usually go to bed at night at** ______

E5-2 **When you are at school, you usually get up in the morning at** ______

E6-1 **Do you have the following sleep disorders?** 0.No 1.Yes E6-1 ☐

E6-2 **Types of sleep disorders (You can choose more than one)**
1.The time to fall asleep is more than 0.5 hours 2.Sleep snoring 3.Open mouth and breathe
4.Suffocate to wake up 5.Sleep apnea 6.Sleepwalking or having nightmares
7.Limb twitching during sleep 8.Excessive sweating 9.Enuresis
10.It is difficult to get up in the morning 11.Feel a headache when you get up in the morning

E7**Time for outdoor activities every day**______days / weeks; An average of ______ hours and ______minutes per day

F. Personal disease history

Types of diseases	Whether or not to suffer from the disease?	Is there any drug treatment at present?
F1 Hypertension	0.Yes 1.No F1	0.Yes 1.No
F2 Type 1 diabetes	0.Yes 1.No F2	0.Yes 1.No
F3 Type 2 diabetes	0.Yes 1.No F3	0.Yes 1.No
F4 Kidney disease	0.Yes 1.No F4	0.Yes 1.No
F5 Heart disease (including congenital heart disease)	0.Yes 1.No F5	0.Yes 1.No
F6 Thyroid disease	0.Yes 1.No F6	0.Yes 1.No
F7 Do you suffer from the following diseases in the past two weeks?	0.Yes 1.No F7	
F8 Types of diseases (multiple choices)	1. Catch a cold<br>2. Fever<br>3. Diarrhea<br>4. Skin trauma and infection.<br>5. Other infectious diseases	

G. Family history of hypertension

	Father	Mother	Grandfather-in-law	Grandmother-in-law	Grandfather	Grandmother
Hypertension?<br>0.Yes 1.No	G1	G2	G3	G4	G5	G6
If there is high blood pressure, how to find it?<br>1. Self-test<br>2. Hospital examination	G7	G8	G9	G10	G11	G12

H. Parents and family situation

	Father	Mother
Age (years)	H1	H2
Height (cm)	H3	H4
Weight (kg)	H5	H6
1. Never smoked.<br>2. Try smoking (even if it's just one or two).<br>3. Recent smoking (at least one cigarette in the past 30 days)	H7	H8
0. Never drank alcohol.<br>1.Try drinking (have drunk at least half a bottle or a can of beer, a small cup of spirits, etc.)<br>2. Drink now (have had at least one glass of wine in the past 30 days)<br>3. Heavy drinking (at least five drinks in 2 hours on at least one day in the past 30 days)<br>4.Drunkenness (symptoms of drunkenness such as dizziness/headache/drowsiness due to drinking too much in the past 12 months)	F9	H10
Degree of education	H11	H12

1.Graduated from junior high school and below
2.Graduated from high school
3.Graduated from junior college
4.Graduated from college
5.Graduate degree

Your family's annual income last year H13

1.<20,000 yuan
2. (2～2.9) ten thousand yuan
3. (4-5.9) ten thousand yuan
4. (6-9. 9) ten thousand yuan
5. (10-14.9) ten thousand yuan
6. 15 ten thousand yuan and above

9.Unknown
The population of your family(resident population) ______ H14 ☐
Fill in the time invest_____ Investigator:____
